# Molecular Diagnostics for Precision Medicine in Colorectal Cancer: Current Status and Future Perspective

**DOI:** 10.1155/2016/9850690

**Published:** 2016-09-06

**Authors:** Guoli Chen, Zhaohai Yang, James R. Eshleman, George J. Netto, Ming-Tseh Lin

**Affiliations:** ^1^Department of Pathology, Penn State College of Medicine, Milton S. Hershey Medical Center, Hershey, PA, USA; ^2^Department of Pathology, Johns Hopkins University School of Medicine, Baltimore, MD, USA

## Abstract

Precision medicine, a concept that has recently emerged and has been widely discussed, emphasizes tailoring medical care to individuals largely based on information acquired from molecular diagnostic testing. As a vital aspect of precision cancer medicine, targeted therapy has been proven to be efficacious and less toxic for cancer treatment. Colorectal cancer (CRC) is one of the most common cancers and among the leading causes for cancer related deaths in the United States and worldwide. By far, CRC has been one of the most successful examples in the field of precision cancer medicine, applying molecular tests to guide targeted therapy. In this review, we summarize the current guidelines for anti-EGFR therapy, revisit the roles of pathologists in an era of precision cancer medicine, demonstrate the transition from traditional “one test-one drug” assays to multiplex assays, especially by using next-generation sequencing platforms in the clinical diagnostic laboratories, and discuss the future perspectives of tumor heterogeneity associated with anti-EGFR resistance and immune checkpoint blockage therapy in CRC.

## 1. Introduction

Colorectal cancer (CRC), predominantly referring to colorectal adenocarcinoma, is one of the most common malignant neoplasms and a leading cause for cancer related deaths worldwide [1]. In 2014, there are nearly 140,000 newly diagnosed patients in the United States where it also ranks in the second place as a cause of cancer related mortality in men and women combined [2]. Therefore, studies aimed at understanding pathogenic mechanisms and optimizing clinical management of CRC have been intensively and devotedly conducted.

In the past two decades, major progress in understanding the genetic alterations of diseases has been achieved and accordingly successful examples of utilizing such information in clinical management are accumulating. These advances have paved the way for the emergence of a new concept, precision medicine, essentially offering individualized medical care to patients based on their unique molecular/genetic profiling and other personalized information. This is in contrast to cohort-based therapy specifically treating patients based on successful therapy of a cohort of similar patients treated previously. In the field of oncology, therapies targeting specific genetic alterations have been proven to be a successful example of practicing precision medicine by significantly improving clinical outcomes compared to conventional chemotherapy and/or radiotherapy. By far, a rapidly growing list of drugs targeting different genetic alterations have been approved by the Food and Drug Administration (FDA) in the United States for treatment of advanced-stage solid tumors [3]. Most of the drugs work through inhibiting kinase activity. For example,* BRAF* inhibitors (vemurafenib and dabrafenib) [4, 5] and* MEK* inhibitor (trametinib) [6] were approved for patients with melanoma bearing* BRAF* p.V600E mutation, anti-EGFR monoclonal antibodies (cetuximab and panitumumab) for CRC without* RAS* mutations [7, 8], EGFR tyrosine kinase inhibitors (gefitinib and erlotinib) targeting certain* EGFR* mutations for non-small-cell lung cancers (NSCLC) [9, 10], and ALK tyrosine kinase inhibitor (crizotinib) for NSCLC carrying the* ALK* gene translocations [11]. Molecular testing of targeted mutations has become essential to select patients for these therapies [12, 13].

To explore more useful “targets” for clinical management of cancers, numerous potential biomarkers have been proposed and investigated with tremendous effort. However, only a limited number of them have so far been proven to be clinically meaningful and subsequently become or potentially become a part of standard patient care. In this review, we focus on the molecular diagnostics currently used in established standard care of CRC, especially those related to targeted therapy or expected to be so shortly.

## 2. Current Guidelines for Targeted Therapy in CRC

In 2009, the American Society of Clinical Oncology (ASCO) issued a recommendation on molecular analysis for* KRAS* gene mutations in patients with metastatic CRC to predict response to anti-EGFR therapy [12]. Following the initial focus on common* KRAS* mutations at codons 12 and 13, recent data have revealed that mutations at codons 59, 61, 117, and 146 and* NRAS* gene mutations are also associated with anti-EGFR resistance [7, 8, 12, 14, 15]. Based on reviews of currently available evidences, ASCO recently updated their provisional clinical opinions: both* KRAS* and* NRAS* exons 2 (codons 12 and 13), 3 (codons 59 and 61), and 4 (codons 117 and 146) (so-called extended* RAS* testing) should be screened for mutations in all patients with metastatic CRC who are candidates for anti-EGFR therapy [16]. Similarly, a provisional guideline from the Association of Clinical Pathologists Molecular Pathology and Diagnostics Group in the United Kingdom also recommends that at least* KRAS* codons 12, 13, 59, 61, 117, and 146 and* NRAS* codons 12, 13, 59, and 61 should be included for molecular analysis in CRC patients [17]. European Society of Medical Oncology and Japanese Society of Medical Oncology recently also revised/updated their clinical guidelines to recommend testing of extended* KRAS/NRAS* mutations [18, 19]. In addition to* RAS*, there is increasing evidence suggesting that the* BRAF* p.V600E mutation makes response to anti-EGFR therapy highly unlikely [7, 20–22]. The Colon/Rectal Cancer Panel from National Cancer Comprehensive Network (NCCN) recently revised its guideline (Version 2.2016) for anti-EFGR therapy by recommending genotyping of tumor tissues in all patients with metastatic CRC for the extended* RAS* mutations as well as* BRAF* mutations (http://www.nccn.org/professionals/physician_gls/f_guidelines.asp). Also, mutations in exon 20 of the* PIK3CA* gene may be associated with anti-EGFR resistance in* KRAS* wild-type cancer [7]. However,* PIK3CA* mutations are often accompanied by a* KRAS* mutation in CRC [7, 23]. The benefit of testing* PIK3CA* mutations to guide anti-EGFR therapy requires further studies with a large cohort of CRC patients carrying wild-type* KRAS* and* NRAS* genes.

In addition to the above guidelines/recommendations largely limited on “what to test” in order to guide oncologists for targeted therapy, more details on “how to test” thus with more practical meaning to molecular diagnostic laboratories and pathologists are also emerging. Using lung cancer as an example, in 2013, the College of American Pathologists (CAP), International Association for the Study of Lung Cancer (IASLC), and Association for Molecular Pathology (AMP) jointly released a guideline regarding molecular diagnostics in lung cancers [24–26]. In addition to clarifying testing for* EGFR *mutations and* ALK* translocations to guide targeted therapy with EGFR or ALK inhibitors, respectively, in all patients with advanced-stage adenocarcinoma, they also offer recommendations and/or expert consensus opinions to address questions such as “how* EGFR* and* ALK* testing should be performed” and “how the molecular testing should be implemented and operationalized.” Soon upon availability, this guideline was endorsed by ASCO [27]. Similarly, a colorectal cancer expert panel from American Society for Clinical Pathology (ASCP), CAP, ASCO, and AMP has drafted a summary of recommendations for guideline on the evaluation of molecular markers for CRC (http://www.amp.org/committees/clinical_practice/CRCOpenComment.cfm). These draft guidelines were opened for comments in 2015. Briefly, they intend to offer “recommendations” and “no recommendations” for questions including not only “which molecular tests should be performed for CRC,” but also “what the appropriate sample for the tests is,” “how testing should be performed,” and so forth. The final version is expected shortly.

## 3. Roles of Pathologists

Currently molecular diagnostics of CRC is largely conducted on tissue specimens embedded in paraffin. The responsibility of surgical pathologists and cytopathologists is not restricted to only making a histological diagnosis. Pathologists, indeed, play crucial roles in preanalytic specimen preparation for molecular diagnostics, including standardizing operating protocols for tissue sampling and processing, requesting mandatory tests, selecting appropriate tissue blocks, and designating adequate areas with sufficient amount and proportion of tumor cells for nuclei acid extraction.

Pathologists also need to estimate tumor cellularity within the designated areas to ensure that the tumor cell percentage is more than the analytic sensitivity (or limits of detection) of the requested molecular assay [28–30]. They also play a critical role in protecting the tissue blocks from unnecessary testing so that critical tests (even future ones) can be performed.

### 3.1. Standard Operating Protocols for Tissue Sampling and Processing

Adequate tissue sampling and processing are critical, not only for histopathological interpretation but also for molecular diagnostics. In our retrospective quality assessment of a next-generation sequencing (NGS) assay, referred specimens experienced a significantly higher failure rate than in-house specimens, presumably due in part to a standardized tissue processing protocol applied to in-house specimens [31]. In this era of precision cancer medicine, pathologists are responsible for revisiting of the standard operating protocols for tissue sampling at the grossing stage and tissue processing in the histopathology laboratories to ensure adequate quality and quantity of nucleic acids for molecular diagnostics. Ten percent neutral-buffered formalin is recommended to fix surgical pathology specimens or cell pellet specimens from fine needle aspiration (FNA) or effusion for 6–48 hours depending on the size of the specimens [25, 26]. Fixative containing heavy metals such as B5 solution or acids such as Bouin's fixative should be avoided if molecular tests are expected to be requested. Bone specimens represent a challenge for molecular diagnosis since bone is common and sometimes the only metastatic site of solid tumors and decalcification step for bone tissues typically using strong acids can damage nucleic acids [32–34]. Therefore, a standard operating protocol for tissue sampling and processing should be established, specifically for bone specimens [35], in the histopathology laboratories to preserve tissues for potential molecular diagnosis.

### 3.2. Selection of Appropriate Blocks and Designation of Adequate Areas for DNA Extraction

In the clinical diagnostic setting, specimens containing low tumor cellularity are not uncommon [36, 37] and may lead to false negative results, particularly for assays with a lower analytic sensitivity, such as Sanger sequencing [38–40]. Assays with a higher analytic sensitivity are preferred for specimens with low tumor cellularity. Nevertheless, selection of appropriate specimens and designation of adequate areas for DNA extraction by the pathologists may be the most cost-effective option for accurate molecular diagnosis [28]. If possible, specimens with a low tumor cell percentage should be avoided, such as areas with a prominent desmoplastic reaction or inflammatory cell infiltration, resected or biopsied specimens of lymph nodes with subcapsular and/or infiltrative metastasis without nodular formation, and FNA specimens with neoplastic cells intermingled with prominent nonneoplastic tissues. Also, regions rich in potential PCR inhibitors such as mucin and necrotic debris should be avoided as well. Prior medical treatment can interfere with tumor tissue adequacy for molecular testing. For example, neoadjuvant therapy for rectal cancers and some high-risk colon cancers can downstage the tumoral lesion; however the accompanied significant depletion of tumor cellularity may lead to false negative results of* RAS* mutations [28, 41, 42]. For patients undergoing neoadjuvant therapy, pathologists may need to seek alternative specimens, such as pretreatment biopsies if available for molecular tests. In addition, tissue blocks from patients with metastatic CRC should be saved for molecular diagnostics, especially those taken by core biopsy or FNA. Assay feasibility of specimens with limited tissues can be improved by limiting block trimming when preparing slides and by avoiding extensive immunohistochemical workups.

### 3.3. Estimation of Tumor Cellularity and Postanalytic Quality Assessment

An accurate estimation of tumor cellularity by pathologists provides a preanalytic measure to ensure specimen adequacy for the analytic sensitivity of the assay. A note should be provided in the report to indicate a potential false result when the tumor cellularity is insufficient. Tumor cellularity also provides an independent parameter for postanalytic quality assessment of the assay [43]. In our retrospective quality assessment of a pyrosequencing assay designed to detect the* BRAF* p.V600E mutation for melanomas, much lower than the expected mutant allele frequencies (equivalent to half of the estimated tumor cellularity assuming a heterozygous mutation) were observed in two specimens. Quality assurance investigation revealed that our original pyrosequencing assay, similar to cobas 4800* BRAF* V600 mutation test, revealed a false, weak p.V600E signal in specimens with a p.V600K mutation. Since the assay was revised, we confirmed that p.V600K and other non-p.V600E mutations are common in melanomas. Correlation between observed and expected mutant allele frequencies may predict tumor heterogeneity and mutant allele-specific imbalance in CRC [36]. A lower than expected mutant allele frequency indicates tumor heterogeneity while a higher than expected mutant allele frequency indicates mutant allele-specific imbalance. We have prospectively and retrospectively confirmed several CRCs with* RAS* mutation present in a subset of tumor cells by analysis of subareas in cases with lower than expected mutant allele frequency. Resistant clones with acquired* RAS* mutations may arise from a small subpopulation present within the original tumor before anti-EGFR therapy or as a consequence of continued mutagenesis over the course of targeted treatment [44]. Presence of* KRAS*-mutant subpopulations may correlate with inferior progression-free survival in CRC patients treated with anti-EGFR therapy [45]. If confirmed, a note should be added in the report to indicate that a* RAS* mutation is present in a subpopulation of the tumor. A note of mutant allele-specific imbalance of the* RAS* gene may also be needed if the information is clinically relevant in the future.

However, assessing tumor cell percentage may not be always precise or accurate [46, 47]. Therefore, a sample with tumor cellularity estimated as borderline adequacy for an assay (e.g., 10–20% of tumor cellularity for a pyrosequencing assay which carries an analytic sensitivity of 10% of tumor cellularity or 5% of mutant allele frequency) should be reevaluated by a second pathologist, preferentially one with both molecular and histological experience. Ideally, pathologists who interpret molecular test results also assess tumor cellularity since they are more aware of the analytic sensitivity of the requested assay. In addition, they can also compare the mutant allele frequency and the estimated tumor cellularity for postanalytic quality assessment of the assay and results as mentioned above. Multiple reasons may account for a perceived discrepancy in correlating the estimated tumor cellularity with the observed mutant allele frequency, one of which is how pathologists determine tumor cellularity. Pathologists are accustomed to using simple linear measurements for microscopic evaluation of sizes. Therefore, they may estimate tumor cellularity by the surface area of tumor cells rather than the ratio of tumor nuclei. This often results in overestimation of tumor cellularity, because cancer cells are commonly larger and visually more impressive than a tiny lymphocyte or stromal cell yet the total DNA amount in both is almost the same.

## 4. Molecular Assays for Targeted Therapy

Since specimens containing low tumor cellularity are common in the clinical diagnostic setting [36, 37], macro- or microdissection of unstained slides or coring of the tissue blocks from the area(s) designated by pathologists has become a routine preanalytic approach to enhance tumor cellularity in many clinical laboratories [29, 30]. However, specimens with a small cluster of tumor cells surrounded by nonneoplastic tissue, such as CRC specimens with prior neoadjuvant therapy, lymph node specimens without formation of distinct metastatic tumor nodules, and cytopathologic specimens with scattered tumor cells are still problematic [28, 48]. In the quality assessment of a NGS assay implemented for clinical mutation detection in CRC, a significant portion of mutations were detected with a low mutant allele frequency [36]. With an analytic sensitivity of 10–20%, Sanger sequencing as the prior gold standard could have missed 8% (with less than 10% mutant allele frequency) or even 23% (with less than 20% mutant allele frequency) of the mutations in this series. Therefore, even with all of the preanalytic efforts, it is still not uncommon to have inadequate or marginally adequate materials. This necessitates assays with higher analytic sensitivity.

### 4.1. Traditional “One Gene-One Drug” Assays

Traditional molecular diagnostics assays usually focus on one mutation or one gene for one drug. Examples include a few assays approved by the FDA of the United States for companion testing of targeted therapy: for example, cobas 4800 BRAF V600 mutation test (for* BRAF* p.V600E mutation), cobas 4800 KRAS mutation test, and therascreen KRAS test (for 7 most common* KRAS* mutations in codons 12 and 13) [49–52]. The cobas 4800 BRAF V600 mutation test, designed specifically for the p.V600E mutation, may also detect non-p.V600E codon 600 mutations at a lower analytic sensitivity. These assays may not be suitable for comprehensive mutational profiling in core biopsy or fine needle aspiration specimens containing limited tissue. A variety of assays have also been applied in molecular diagnostics laboratories to detect* KRAS* or* BRAF* mutations with a limit of detection ranging from 10–20% mutant allele for Sanger sequencing, approximately 5% for pyrosequencing and high-resolution melting curve analysis, to 1–5% for real-time PCR-based assay [53–57]. Results from different assays are usually concordant except for specimens with poor tumor cellularity for which more sensitive assays are needed to prevent false negative results. In addition to molecular assays, immunohistochemistry (IHC) stain is also a highly sensitive and cost-effective assay for detection of* BRAF* mutation [58, 59]. IHC stain is especially valuable for specimens with scattered tumor cells intermixed with abundant nontumor cells. However, monoclonal antibodies against the BRAF p.V600E protein adopted in current IHC assays do not detect other mutations with adequate analytic sensitivity and specificity.

### 4.2. Multiplex Assays

With continuous expansion of predictive markers for targeted therapeutics, molecular testing has been in a transition from the traditional “one test-one drug” model to a multiplex genotyping platform to simultaneously test a panel of genes for a specific cancer [60, 61]. Primer extension-based multiplex assays, such as the multiplex SNaPshot assay or the Sequenom MassARRAY system, are capable of testing multiple targets in a single reaction while retaining an analytic sensitivity of 5% or below [62, 63]. More excitingly, massively parallel sequencing or NGS technology has revolutionized genome research and also will soon become the most cost-effective multiplex sequencing platform in the clinical diagnostic setting as more and more biomarkers join standard patient care [60]. The US Centers for Disease Control and Prevention (CDC) has convened the Next-Generation Sequencing: Standardization of Clinical Testing (Nex-StoCT) workgroup and published guidelines to address the 4 components of quality management of NGS assays in clinical laboratories: test validation, quality control, proficient test, and reference materials [64]. The 6 analytic performance characteristics for clinical validation of NGS assays were also defined. Although the workgroup focused on heritable genetic diseases, the same principles can also be applied to precision cancer medicine. Subsequently, the workgroup (Nex-StoCT II) also published recommendations to design, optimize, and implement informatics pipelines for clinical NGS assays [65]. In 2015, CAP issued laboratory standards for NGS clinical tests including the new checklist requirements [66].

### 4.3. Clinical Validation and Implementation of NGS Platforms

Mutational profiling of cancer specimens based on NGS assays has been validated and implemented for prospective standard patient care or clinical trial in the clinical molecular diagnostic laboratories. The spectrum of mutations examined (reportable range) ranges from a panel of genes for a specific tumor or a group of tumors [67–70] to a larger panel of targetable/actionable genes or oncogenes/tumor suppression genes [37, 71] and whole exome sequencing [72, 73]. Currently, treatment guided by the comprehensive analysis of whole exome sequencing is still limited in a small fraction of patients. The ability to generate large amounts of data of unproven significance, therefore, should not take precedence over the timely generation of clinical useful data. When an extensive NGS panel is offered for clinical diagnosis, it is recommended that an assay with a shorter turnaround time is reported first, followed by a more comprehensive assay which may take longer time to complete [24–26].

We have validated and implemented a NGS platform using the AmpliSeq Cancer Hotspot Panel and Personal Genome Machine in a* Clinical Laboratory Improvement Amendments-* (CLIA-) certified laboratory [67]. In a retrospective quality assessment study, we surveyed the performance characteristics of the NGS assay conducted in 310 CRC specimens [36]. NGS demonstrated a high analytic sensitivity (2% or lower mutant allele frequency), broad reportable ranges of mutation spectrum relevant to anti-EGFR therapy in* KRAS*,* NRAS*,* BRAF,* and* PIK3CA* genes, capacity for quantitative measurement of mutant allele frequencies, and simultaneous detection of concomitant mutations [36]. The test feasibility was approximately 98% (2% of combined rejection rate and failure rate) [31] with a turnaround time of 3–6 working days for 90% or more of specimens as the assay was conducted twice a week (unpublished data). Seventeen percent of* KRAS* mutations were outside codons 12 and 13 and 48% of* PIK3CA* mutations were outside the 3 most common mutated codons 542, 545, and 1047. The incidence of tumors with predicted resistance to anti-EGFR therapy increased as reportable ranges became wider, from 40% if only mutations in* KRAS* exon 2 were tested to 47% if exons 2–4 were included, 48% if* KRAS* and* NRAS* exons 2–4 were included, 58% if also including* BRAF* codon 600 mutations, and 59% if adding* PIK3CA* exon 20 mutations. Interestingly, right-sided CRCs were found with a higher risk of predicted anti-EGFR resistance. The advantage with broader reportable ranges may also be helpful in elucidating the clinical significance of uncommon mutations [36, 74]. For example, we identified* BRAF* mutations with reduced or silent kinase activity, such as mutations affecting codon 594. CRC patients with kinase-impaired* BRAF* mutations may respond to anti-EGFR therapy [7]. Kinase-impaired* BRAF* mutants showed a significantly higher incidence of concomitant activating* KRAS* or* NRAS* mutations [36, 75]. In the presence of oncogenic RAS proteins, kinase-impaired BRAF forms a complex with CRAF, which leads to hyperactivation of the CRAF/MEK/ERK cascade [76, 77]. Therefore, CRC patients with coexisting kinase-impaired* BRAF* mutation and activating* RAS* mutation may benefit from MEK inhibitors [75]. 

## 5. Future Perspectives

With the inspiration of achieved success in precision cancer medicine, tremendous efforts have been devoted to discovering more biomarkers for potential usage in clinical diagnosis, prediction, and prognostication of CRC [78]. In addition to mutations within the mitogen-activated protein kinase (MAPK or RAS/RAF/MEK/ERK) pathway and the phosphatidylinositol 3-kinase (PI3K/AKT/mTOR) pathway, the potential molecular markers also include epigenetic alterations and microRNA expression. Also, exploration of “old” markers for new utilization is feasible and economical given that a single marker can be used for multiple purposes or in different situations. For example,* BRAF* mutation status has several clinical implications in CRC. Detecting the p.V600E mutation has been a part of the algorithm to distinguish sporadic CRC with microsatellite instability (MSI) from hereditary nonpolyposis colorectal cancers (HNPCC or Lynch syndrome) since this* BRAF* mutation is present only in sporadic cases [79, 80].* BRAF* mutations along with MSI status can also offer information for risk stratification of CRC. Patients with a p.V600E mutation may carry an inferior prognosis in microsatellite-stable (MSS) CRC but not CRC with MSI [81, 82]; however, a study suggests that in advanced (metastatic) disease patients with a p.V600E mutation carry an inferior prognosis also in MSI CRC [83]. MSI status also has multiple implications. Besides the well-known role in screening for HNPCC, MSI is associated with a lower stage of CRC at diagnosis and a favorable stage-specific prognosis [84], although conflicting results in stage IV patients exist [85]. In terms of predictive significance, MSI patients may not benefit from 5-FU based adjuvant therapy [86–88], though benefit has been observed in stage III patients with suspected HNPCC [89]. Further studies are needed to clarify the predictive value of MSI status [90]. More importantly, a recent clinical trial demonstrated the utility of MSI status as a predictive marker for responsiveness to PD-1 blockade immunotherapy in advanced CRC patients [91]. As expected, there are many ongoing clinical trials regarding targeted therapy, immunotherapy, and combinatorial therapy for CRC patients. Undoubtedly there will be prosperous progress of precision medicine in CRC in the near future. For the rest of this review, we will focus on recent advance in the field of tumor heterogeneity associated with anti-EGFR resistance and immunotherapy.

### 5.1. Acquired Resistance to Anti-EGFR Therapy

Resistance to targeted therapy can be classified into intrinsic (primary) or acquired (secondary) resistance. Intrinsic resistance is usually defined as immediate failure of treatment, whereas acquired resistance is defined as the disease progression following a period of clinical response. By far, best studied is the resistance to EGFR tyrosine kinase inhibitors in lung cancers [92]. In additional to testing for* EGFR* mutations for first-line targeted therapy, examination of the most prevalent acquired resistance mutation,* EGFR* p.T790M, has become a common clinical practice to select patients for third-generation tyrosine kinase inhibitors [92]. The whole picture of acquired resistance mechanisms in CRC was not fully understood until the last few years, presumably because of lack of second-line targeted therapy to overcome acquired resistance and concern of risk for tissue biopsy. Mutational profiling of cell-free circulating tumor DNA (ctDNA) in the blood (so-called liquid biopsy) provided an alternative noninvasive approach to uncover the genetic landscape of acquired resistance mechanisms in CRC [44, 93–96].

Several mechanisms underlying acquired anti-EGFR resistance in CRC have been reported [97–99]. While mutations responsible for acquired resistance to small molecule kinase inhibitors often occur within the kinase domain, there is significant overlap between the intrinsic and acquired genomic alterations leading to anti-EGFR resistance in CRC. These include mutations in the* KRAS*,* NRAS*,* BRAF*, and* MAP2K1* within the MAPK pathway [97–99], mutations involving codons 464, 465, 467, 491, 492, and 494 within the extracellular domain [93, 100, 101] and codons 714 and 794 within the kinase domain of EGFR [102], and amplification of the* KRAS*,* MET*, and* EBRR2* genes [93–95]. Acquired resistance mutations commonly involve codons 12, 13, and 61 of the* KRAS* gene and codon 61 of the* NRAS* gene in contrast to codons 12 and 13 of the* KRAS* gene in the primary resistance setting. The reported acquired* EGFR* mutations were rare or not seen in CRCs prior to anti-EGFR therapy [103]. Recently, whole exome sequencing accompanied with in vitro study of anti-EGFR sensitivity has recognized, albeit infrequently, mutations in the* ERBB2*,* EGFR*,* FGFR1*,* PDGFRA*, and* MAP2K1* as potential mechanisms underlying primary resistance as well as the tyrosine receptor adaptor gene,* IRS2*, in tumors with increased sensitivity to anti-EGFR therapy [102].

### 5.2. Tumor Heterogeneity Associated with Anti-EGFR Resistance

A phenomenon relevant to drug resistance is tumor heterogeneity [104, 105]. Comprehensive analysis by NGS has revealed remarkable genetic variation between and within tumors. Genomic heterogeneity seen in tumors prior to or after targeted therapy poses a major challenge to precision cancer medicine. Tumor heterogeneity associated with primary or acquired anti-EGFR resistance has been well documented in lung cancers. Presence of the* EGFR* p.T790M mutation in a minor subpopulation of tyrosine kinase inhibitor-naïve tumors predicts an inferior response to the first-line EGFR tyrosine kinase inhibitors [106, 107]. Some of the best evidence of tumor heterogeneity associated with acquired resistance in lung cancers is the reciprocal relationship between the* EGFR* p.T790M mutation and transformation of small cell carcinoma. In patients with multiple resistant metastases showing separate adenocarcinoma and small cell carcinoma components, p.T790M was often detected only in the adenocarcinoma component [108, 109], while small cell carcinoma component carried* RB1* mutations, but not p.T790M [108–110].

As mentioned previously, CRC with “acquired”* RAS* mutations may arise from a small subpopulation present within the original tumor before anti-EGFR therapy or as a consequence of continued mutagenesis over the course of targeted treatment [44]. Tumor heterogeneity associated with intrinsic* KRAS* mutations carries an inferior response to anti-EGFR therapy [45]. The observation of multiple acquired resistance mutations in plasma ctDNA from the same patients also suggested tumor heterogeneity associated with acquired anti-EGFR resistance in CRC [44, 93, 94, 96]. Recently, Russo et al. confirmed that tumor heterogeneity associated with acquired anti-EGFR resistance may affect lesion-specific response to the second-line targeted therapy and that plasma ctDNA is a better source of specimens for comprehensive capture and dynamic monitoring of resistance mutations than tissue specimens which are subjected to risk and spatial selection bias of a core biopsy [99].

Strategies have been proposed to overcome acquired resistance associated with tumor heterogeneity. One potential strategy is to combine anti-EGFR therapy with MEK or ERK inhibitors [94, 97]. Although tumor heterogeneity may lead to metastatic lesions demonstrating different resistance mechanisms, these mutations are biologically convergent on the MAPK pathway to sustain activation of MEK and ERK. This was supported by a study of ctDNA showing one or more acquired resistance mutations in the genes involved in the MAPK pathway in 23 of 24 patients [96]. Another potential strategy is to identify intrinsic resistance mutations associated with tumor heterogeneity by using ultrasensitive assays to examine plasma ctDNA. Preclinical studies have shown that combining anti-EGFR therapy with other kinase inhibitors may be effective in tumors harboring mutations or amplification in* ERBB2*,* MET*,* EGFR*,* FGFR1*, and* PDGFRA* genes [93, 102].

### 5.3. Anti-PD-1 Immunotherapy in CRC Patients

Immune checkpoint blockage inhibition has provided an alternative option for treatment of metastatic solid tumors [111–115]. In particular, antibody-mediated blockade of programmed death 1 (PD-1) or programmed death ligand 1 (PD-L1) can induce durable disease-free survivals, albeit only in a portion of patients with advanced melanomas, lung cancers, and bladder cancers [114–116]. Several clinical trials targeting immune checkpoint have also been conducted in gastrointestinal cancers, including CRC [117]. In 2015, Le and colleagues reported that MSI is a marker to predict the benefit of pembrolizumab, an anti-PD-1 immune checkpoint inhibitor, in CRC and other solid tumors [91]. These results are consistent with the pathogenesis of MSI in hereditary or sporadic CRC with defect in the mismatch repair machinery. Defective mismatch repair causes hypermutation of the genome, including MSI, and generates tumor neoantigens [118, 119]. CRC with MSI also demonstrated highly upregulated expression of multiple immune checkpoint molecules, including PD-1 and PD-L1 [119]. These may explain the profound lymphocyte infiltration as well as better outcomes and response to anti-PD-1 monoclonal antibody in CRCs with MSI [91, 120, 121].

### 5.4. Predictive Biomarkers for Immune Checkpoint Blockage Therapy

Currently, researchers have been aggressively exploring potential markers to select candidates who will benefit from immune checkpoint blockage therapy. These include expressional levels and gene amplification of PD-L1 [116, 122–124], MSI status [91], and genomic hypermutation [91, 111, 125, 126]. Although strong PD-L1 expression has been reported in a subset of CRCs [127, 128] and appears to predict repose to anti-PD-L1 therapy [116, 122–124], the definition of PD-L1 positive CRC needs further standardization and validation in a clinical diagnostic setting. In addition, tumors in response to immune checkpoint blockage may not have strong PD-L1 expression [117]. MSI test is a routine assay in most molecular diagnostics laboratories for screening HNPCC. The revised NCCN guideline (version 2.2016) has suggested screening for HNPCC (Lynch syndrome) in CRC patients aged 70 year or younger and those older than 70 who meet the Bethesda guidelines (http://www.nccn.org/professionals/physician_gls/f_guidelines.asp). Further studies are warranted to elucidate if the MSI assay or immunohistochemical stain of the mismatch repair machinery components (MLH1, MSH2, MSH6, and PMS2) may become a standard of care for those solid tumors with a higher incidence of MSI, such as CRC and endometrial cancer [129].

Mutation load may prove to be the most important predictor for immune checkpoint blockage therapy [91, 111, 125, 126]. MSI occurs in a subset of hypermutated tumors with defective mismatch repair machinery. There are many mechanisms causing a mutator phenotype with hypermutations, such as exposure to external mutagens (e.g., cigarette smoke and UV radiation), endogenous mutagens (e.g., reactive oxygen species), and mutations in the* POLE* or* POLD1* genes encoding the DNA polymerases [130]. Tumors with hypermutation caused by these alternative mechanisms are anticipated to have enhanced sensitivity to checkpoint blockade. Germline or somatic mutations in the regions encoding exonuclease domain of POLE and POLD1 impair polymerase proofreading and lead to an exceedingly high rate of base substitution mutations [131–133]. Results from the Cancer Genome Atlas showed hypermutation in 16% of CRC, including three-quarters with MSI and one-quarter with* POLE* mutations [134]. A recently study [135] investigating association between* POLE* mutations and prognosis in more than 4500 stage II/III CRC patients shows the pathogenic somatic* POLE* mutations were detected in approximately 1.0% of CRCs.* POLE* mutations were mutually exclusive with MSI. Compared with MSS* POLE* wild-type CRCs,* POLE*-mutant CRCs showed increased CD8+ lymphocyte infiltration and expression of cytotoxic T-cell markers and effector cytokines, with a level similar to that observed in immunogenic MSI tumors. Both* POLE* mutations and MSI status were associated with significantly reduced risk of recurrence compared with MSS CRCs in multivariable analysis. Higher* POLE* mutation rates (7–12%) have also been reported in endometrial cancers [136, 137], similarly characterized by a robust intratumoral T-cell response [138], and carry an excellent outcome [136, 137].* POLE* mutations in CRC, though uncommon, may be associated with a favorable response to anti-PD-1 or anti-PD-L1 immunotherapy. However, the excellent prognosis demonstrated in this group of patients also underscores the importance of POLE mutations in precision medicine.

In summary, MSI is a routine clinical assay not only being suitable for screening for HNPCC but also being potentially predictive for immune checkpoint blockage therapy. However, testing for MSI status picks up only those CRCs with defective mismatch repair machinery, but not other hypermutated tumors. Whole exome sequencing is certainly a robust approach to define hypermutation. Currently, it may not be practical to perform whole exome sequencing as a daily clinical routine to select patients for immune checkpoint blockage therapy. Alternatively, NGS assays may be designed to include a panel of genes to identify tumors with a high mutation load for potential treatment with immunotherapies as well as driver mutations for targeted therapy [139]. Further clinical trials are also needed to guide through the complexity in selecting targeted therapeutic agents in combination or in sequence with immune checkpoint blockage inhibitors [140].

## 6. Conclusion

The established and/or potential key molecular markers in molecular diagnosis of CRC are briefly summarized in Table 1. Current guidelines from several organizations recommend testing extended* RAS* genes to select CRC patients for anti-EGFR targeted therapy. The* BRAF* p.V600E mutation is also highly likely to predict for anti-EGFR resistance. More studies are needed to elucidate the role of* PIK3CA* mutations. The convergence of recent advances in molecular technology and rapid expansion of targeted therapeutics is transforming the approach in clinical molecular diagnostic laboratories from the traditional “one test-one drug” paradigm to the multiplexed genotyping platform, especially NGS. In the clinical diagnostic setting, NGS assays demonstrate a high sensitivity, a broad reportable range, and a precise measurement of mutant allele frequency. Eventually, NGS will become the most cost-effective assay as more and more genetic alterations have demonstrated clinical utility. In this era of precision cancer medicine, pathologists play crucial roles in molecular diagnostics. They are responsible for preserving tumor tissues with adequate quantity and quality of nucleic acids for molecular tests. Tumor cellularity estimated by pathologists provides an important parameter for postanalytic quality assessment of the assays and results. Recently studies have identified more potential intrinsic resistance mutations and uncovered the landscape of acquired resistance mechanisms. Tumor heterogeneity associated with anti-EGFR resistance poses challenges to targeted therapeutics in CRC. Strategies have been proposed to overcome anti-EGFR resistance resulting from tumor heterogeneity. Immune checkpoint blockage therapy has emerged as a critical alternative option for metastatic cancers with durable progression-free survival, although long term follow-up has not been achieved. In addition to the MSI test, further efforts to develop assays amenable in clinical molecular diagnostic laboratories are warranted to select CRC patients for targeted therapy and/or immunotherapy.

## Figures and Tables

**Table 1:** Molecular diagnostics markers of CRC currently used in established standard care or potentially being used in the near future.

Clinical utility	Markers^*∗*^
Diagnostic markers	*BRAF *[79, 80]; MSI [129]

Predictive markers	
(i) Primary resistance to anti-EGFR mAb	*KRAS* and *NRAS* [7, 8, 12, 14–19]; *BRAF *[7, 20–22]; *PIK3CA* [7]; other potential markers (*ERBB2*, *EGFR*, *FGFR1*, *PDGFRA,* and *MAP2K1*) [102]
(ii) Secondary resistance to anti-EGFR mAb	Mutations in the MAPK pathway (*KRAS*, *NRAS*, *BRAF, *and *MAP2K1*) [97–99];* EGFR* [93, 100–102]; amplification of *KRAS*; *MET; ERBB2* [93–95]
(iii) Immune checkpoint blockage therapy	PD-L1 expression [116, 122–124]; MSI [91]; genomic hypermutation [91, 111, 125, 129]

Prognostic markers	*BRAF* [81–83]; MSI [84, 85]; *POLE* [135]

^*∗*^The numbers in the parentheses indicate the references cited in this article.
